# On a new generalized lindley distribution: Properties, estimation and applications

**DOI:** 10.1371/journal.pone.0244328

**Published:** 2021-02-24

**Authors:** Ali Algarni

**Affiliations:** Department of Statistics, Faculty of Science, King AbdulAziz University, Jeddah, Saudi Arabia; Tongii University, CHINA

## Abstract

In this study, an extension of the generalized Lindley distribution using the Marshall-Olkin method and its own sub-models is presented. This new model for modelling survival and lifetime data is flexible. Several statistical properties and characterizations of the subject distribution along with its reliability analysis are presented. Statistical inference for the new family such as the Maximum likelihood estimators and the asymptotic variance covariance matrix of the unknown parameters are discussed. A simulation study is considered to compare the efficiency of the different estimators based on mean square error criterion. Finally, a real data set is analyzed to show the flexibility of our proposed model compared with the fit attained by some other competitive distributions.

## 1 Introduction

Recently, many researchers have suggested new generalization for life time distributions used in statistics and possess flexibility in applications. Although the wide range of applications of the Lindley distribution [1] has a wide range of applications, it does not provide a good fit for modeling phenomenon with non-monotone failure rates, such as bathtub upside down failure shaped. For this lack of flexibility, many authors proposed a new generalizations of the traditional Lindley distribution by adding one or more shape parameters to add more flexibility to the PDF and the hazard rate function. Extended generalized Lindley (EGL) distribution is a very important lifetime and survival distribution which can be used as an effective alternative to the well known distributions such as generalized Lindley (GL), Lindley (L) and exponential distributions. It has different applications in modelling various types of data including economics and actuarial sciences data because its hazard rate can be increasing, decreasing, upside down bathtub shaped and unimodal. In addition, this model presented a better fit to data resulting in accurate results and predictions, which should facilitate better public policy in a wide range of areas including medicine, genetics, environmental health, reliability, survival analysis and actuarial sciences data because its hazard rate can be increasing, decreasing, upside down bathtub shaped and unimodal. Several types of lifetime model distribution have been proposed in literature. Zakerzadah and Dolati [2] presented GL distribution and studied its statistical properties and applications. Also, Oluyede and Yang [3] introduced a new class of GL distributions with applications. Nadarajah et al. [4] introduced GL distribution with shape and scale parameters *γ*, λ, respectively, the probability density function (PDF) is given by

$$\begin{array}{c} f\left(x\right)=\frac{\gamma \lambda^{2}}{1+\lambda }\left(1+x\right)e^{-\lambda x}{\left[1-\frac{1+\lambda +\lambda x}{1+\lambda }e^{-\lambda x}\right]}^{\gamma -1},\;\;x>0,\;\left(\lambda ,\gamma >0\right) \end{array}$$ (1)

and cumulative distribution function (CDF) is

$$\begin{array}{c} F\left(x\right)={\left[1-\frac{1+\lambda +\lambda x}{1+\lambda }e^{-\lambda x}\right]}^{\gamma },\;\;x>0,\;\left(\lambda ,\gamma >0\right). \end{array}$$ (2)

On the other hand, Marshall and Olkin [5] proposed a method of adding a new shape parameter to any well-known distribution whose cdf denoted by *F*(*x*), as follows

$$\begin{array}{c} G(x;\delta )=\frac{F(x)}{1-\left(1-\delta \right)\bar{F}\left(x\right)},\;x\in R,\delta >0,\bar{\delta }=1-\delta . \end{array}$$ (3)

where *δ* > 0. Many new distributions have been proposed in the literature by considering *F*(*x*) to be normal distribution by Ghitany et al. [6], Birnbaum-Saunders distribution by Lemonte [7]. The Marshall- Olkin (M-O) extended distributions have an interesting failure rate function facilitating its use in modeling real situations in a better manner than the basic distribution. For more details see Cordeiro and Lemonte [8], Okasha and kayid [9] and Okasha and Al-Shomrani [10]. The supplemental parameter *δ* involved in the transformed distribution described in Eq (3) is called the “tilt parameter”. In fact, the failure rate functions *h*(*x*) and *r*(*x*) corresponding to the transformed distribution and the initial distribution are such that, for all *x* ≥ 0, one has *h*(*x*)≤*r*(*x*) if *δ* > 1 and *h*(*x*)≥*r*(*x*) if 0 < *δ* ≤ 1. This means that the failure rate of the new distribution is shifted below (respectively above) when *δ* > 1 (respectively when 0 < *δ* ≤ 1). Many authors used Marshall and Olkin’s (1997) method to generate a new continuous distribution by taking the baseline *F*(*x*) of any known distribution. Okasha et al. [11] introduced a detailed study of M-O Extended inverse Weibull which can be obtained as a mathematical propery with estimation of the maximum Likelihood and stress-strength parameter. Benkhelifa [12] also proposed properties and applications for the M-O extended generalized Lindley distribution. Okasha and Shrahili [13] obtained various results on the M-O Burr type XII Distribution in the context of reliability properties and survival analysis. In this paper we propose a new extension of the GL distribution called the M-O Extended Generalized Lindley Distribution and study some of its properties. The present work is organized as follow: (1) definition of the probability density function, cumulative distribution function and survival function of the EGL distribution. (2) presentation of the obtained values of some properties of the new distribution such as (reversed) failure rate, (reversed) mean residual lifetime, quantiles, moments, order statistics and stochastic ordering. (3) discussion of maximum likelihood estimates (MLEs) and asymptotic confidence intervals from the Fisher information matrix (FIM) of the model parameters. (4) An application of the extended distribution to waiting times (in minutes) before service of 100 bank customers is given showing that the present model provides a better fit to the real data than some other known distributions. Finally, conclusions and remarks of the current and future research are presented.

## 2 New family and its own sub-models

This section proposes the new family distribution and derives density and survival functions from this family.

### 2.1 New family description

Let Λ = (λ, *γ*, *δ*) and inserting Eq (2) in Eq (3), a new distribution denoted as EGLD (*x*;Λ) can be obtained. Then, the CDF of the EGLD can be obtained as;

$$\begin{array}{c} G(x;\Lambda )=\frac{\kappa }{1-\bar{\delta }(1-\kappa )},\;x>0,\;\Lambda >0, \end{array}$$ (4)

where $\kappa ={\left(1-\frac{\left(\lambda +\lambda x+1\right)e^{-\lambda x}}{\lambda +1}\right)}^{\gamma }$.

The corresponding survival function (SF) and the PDF are defined by

$$\begin{array}{c} \bar{G}(x;\Lambda )=\frac{\delta (1-\kappa )}{1-\bar{\delta }(1-\kappa )},\;x>0,\;\Lambda >0, \end{array}$$ (5)

and

$$\begin{array}{c} g\left(x;\Lambda \right)=\frac{\lambda^{2}\gamma \delta }{\lambda +1}(\frac{\kappa^{1-\frac{1}{\gamma }}}{{\left(\delta +\bar{\delta }\kappa \right)}^{2}})\left(x+1\right)e^{-\lambda x}.\;x>0.\;\Lambda >0, \end{array}$$ (6)

respectively. The next proposition presents the behavior of the pdf of the EGLD (*x*;Λ) with various choices of parameters.

**Proposition 2.1**. *Let X ∼ EGLD* (*x*;Λ), *then*:

1. *If γ* < 1, *then X has a decreasing pdf*.
2. *If γ* ≥ 1, *then X has an increasing pdf*.
3. *If γ* ≥ 1, *then X upside-down bathtub shaped*.

**Fig 1:**
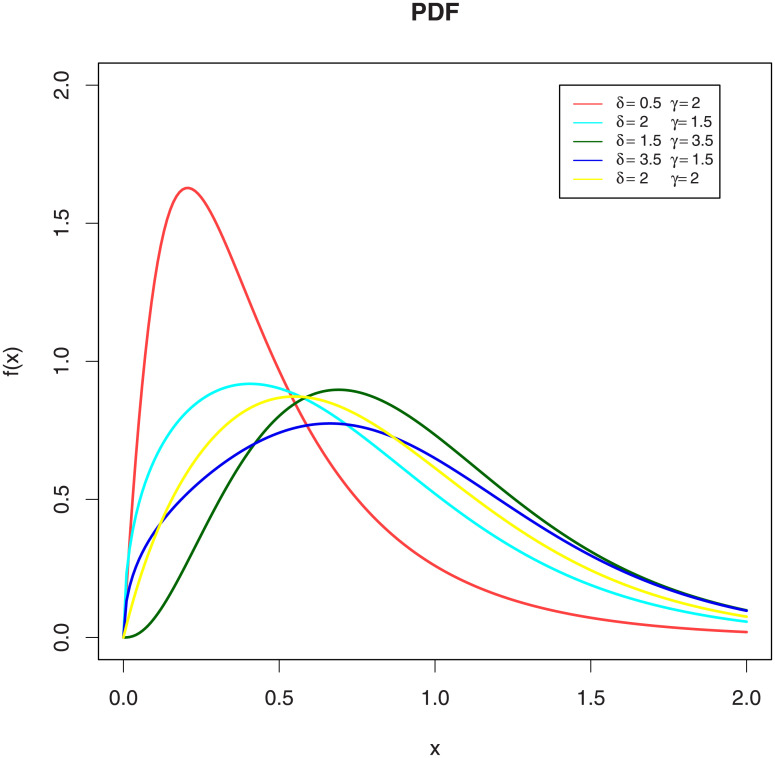
Plots PDF of the EGLD with λ = 2.9. Figure (1) shows the various shapes of the PDF of the EGLD given by Eq (6) by choosing the shape parameter, λ, to be 2.90 in all the cases and different values of the shape parameters. Figure (1) indicates that the proposed distribution is suitable to model the right skewed data.

Fig (1) shows the various shapes of the PDF of the EGLD given by Eq (6) by choosing the scale parameter, λ, to be 2.90 in all the cases and different values of the shape parameters. Fig (1) indicates that the proposed distribution is suitable to model the right skewed data.

### 2.2 Special models of the new family

The next example shows that the new family contains the GL and L distributions as special cases.

**Special cases 1**. *Let X* ∼ *EGLD*(*x*;Λ), *then*

1. *If δ* = 1 *in* Eq (6), *then X* ∼ *GLD*(*x*;λ, *γ*).
2. *If δ* = *γ* = 1 *in* Eq (6), *then X* ∼ *LD*(*x*;λ).

## 3 Reliability and statistical properties

In this section, reliability and some statistical properties of the EGLD are presented, especially quintile function, moments, (reversed) failure rate, mean residual life, order statistics and stochastic orderings.

### 3.1 Failure rate and mean residual life

Let *T* ≥ 0 be a continuous random variable with cdf *F*(*t*) and pdf *f*(*t*), the failure rate (FR) function of the EGLD is defined as

$$\begin{array}{c} h\left(t\right)=\lim_{\Delta t\to 0}\frac{P(T<t+\Delta t|T>t)}{\Delta t}=\frac{g(t)}{\bar{G}\left(t\right)}. \end{array}$$ (7)

For the EGLD, the failure rate function *h*(*t*) is

$$\begin{array}{c} h\left(t;\delta \right)=\frac{\lambda^{2}\gamma }{\left(\lambda +1\right)}\frac{\left(t+1\right)\kappa^{\gamma -1}e^{-\lambda t}}{\left(\kappa^{\gamma }-1\right)\left(\delta \left(\kappa^{\gamma }-1\right)-\kappa^{\gamma }\right)}, \end{array}$$ (8)

where

$$\begin{array}{c} \kappa =1-\frac{\left(\lambda +\lambda t+1\right)e^{-\lambda t}}{\lambda +1} \end{array}$$ (9)

**Proposition 3.1**. *Let h*(*t*) *be the failure rate function of a random variable T distributed according to EGLD* (Λ). *Then*

1. *h*(*t*) *is increasing for* λ < 1 *and γ* > 1.
2. *h*(*t*) *is bathtub shaped for* λ > 1 *and γ* > 1.
3. *h*(*t*) *is decreasing for* λ < 1 *and γ* < 1.

The mean residual life (MRL) can be obtained by general formula (see Navarro et al. [14])

$$\begin{array}{c} M_{{}_{T}}(t)=E(T-t|T>t)=\frac{1}{\bar{G}(t)}\int_{t}^{\infty }\bar{G}\left(x\right)dx.\;t>0. \end{array}$$ (10)

**Lemma 3.2**. *Let T* ∼ *EGLD* (*t*;Λ), *then the MRL function of a lifetime random variable is given by*:

$$\begin{array}{c} M_{{}_{T}}(t)=\frac{\left(\delta -1\right)\kappa^{\gamma }-\delta }{\delta (\kappa^{\gamma }-1)}E\left(t;\lambda ,\gamma ,\delta \right), \end{array}$$ (11)

*where*

$$\begin{array}{ccc} E(t;\Lambda ) & = & \frac{\delta (1+\lambda )}{\lambda }\sum_{k,i,j=0}^{\infty }(\begin{array}{c} k+1\\ j \end{array})(\begin{array}{c} \gamma j\\ i \end{array})\frac{{\left(-1\right)}^{j}{\left(1-\delta \right)}^{k}e^{i(1+\lambda )}}{{\left(i\left(1+\lambda \right)\right)}^{i+1}}\\ & & \times \Gamma (i+1,i(1+\lambda +\lambda t)), \end{array}$$ (12)

*and*

$$\begin{array}{c} \Gamma \left(a,y\right)=\int_{y}^{\infty }y^{a-1}e^{-y}dy. \end{array}$$ (13)

*be the upper incomplete gamma, for more details, see Wall* [15].

**Fig 2:**
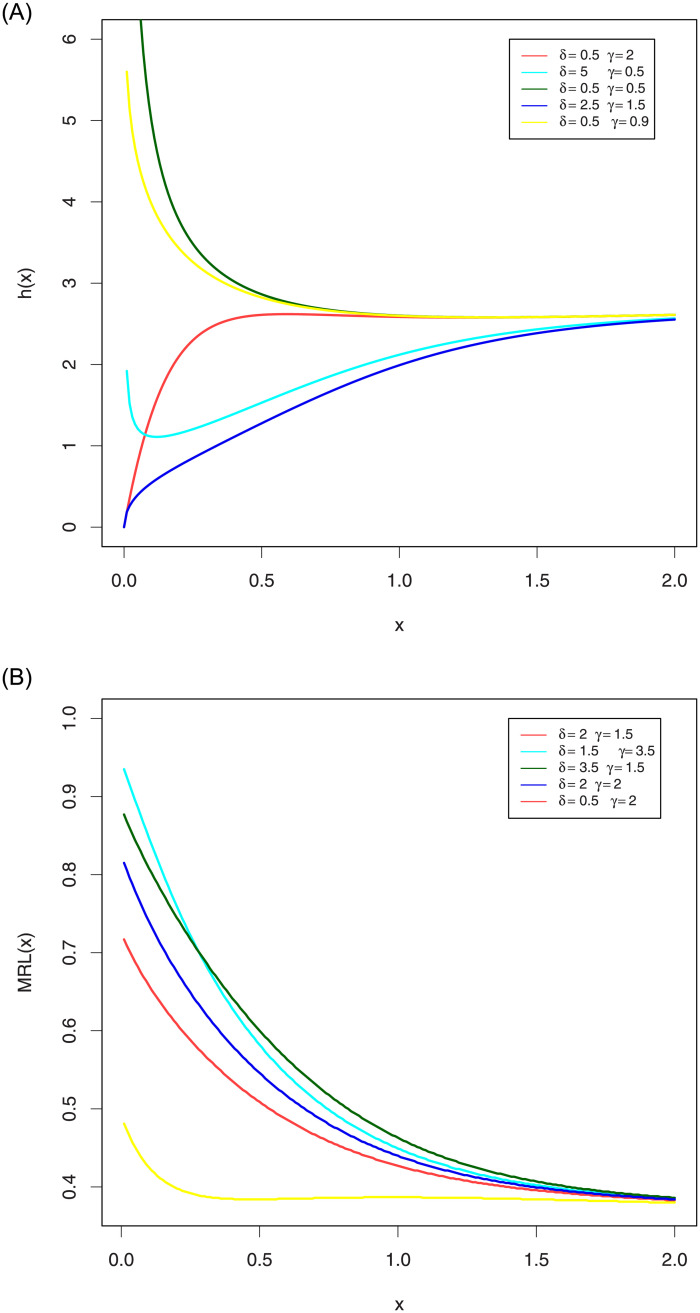
Plots FR and MRL of the EGLD with λ = 2.9. Figure (2) shows the different shapes of its FR and MRL for some selected parameters values with scale parameter one. This Figure indicates that the EGLD FR can be monotonically increasing and MRL can be monotonically decreasing.

Fig (2) shows the different shapes of its FR and MRL for some selected parameters values with scale parameter one. This Figure indicates that the EGLD FR can be monotonically increasing and MRL can be monotonically decreasing.

### 3.2 Reversed failure rate and mean inactivity time

For a continuous distribution with pdf, *g*(*t*), and CDF, *G*(*t*), the failure rate function, also known as the reversed failure rate (RHR) function, is defined as

$$\begin{array}{c} r\left(t\right)=\lim_{\Delta t\to 0}\frac{P(T>t-\Delta t|T\leq t)}{\Delta t}=\frac{g(t)}{G(t)}. \end{array}$$ (14)

For the EGLD, the reversed failure rate function *r*(*t*) is

$$\begin{array}{c} r\left(t;\delta \right)=\frac{\lambda^{2}\gamma \delta }{\left(\lambda +1\right)}\frac{\left(t+1\right)e^{-\lambda t}}{\kappa (\bar{\delta }\kappa^{\gamma }+\delta )}. \end{array}$$ (15)

For a continuous distribution with pdf *g*(*t*) and cdf *G*(*t*), the mean inactivity time (MIT) function is defined as

$$\begin{array}{c} m\left(t\right)=\frac{1}{G(t)}\int_{0}^{t}G\left(x\right)dx,\;t>0. \end{array}$$ (16)

For the EGLD, the mean inactivity time function *m*(*t*) is

$$\begin{array}{ccc} m(t) & = & \frac{\kappa^{\gamma }}{1-\left(1-\delta \right)\left(1-\kappa^{\gamma }\right)}D\left(t;\lambda ,\gamma ,\delta ,1\right), \end{array}$$ (17)

where

$$\begin{array}{ccc} D(t;\Lambda ,a) & = & \sum_{k,i,j=0}^{\infty }(\begin{array}{c} k\\ j \end{array})(\begin{array}{c} \gamma (j+1)-1\\ i \end{array}){\left(\frac{\lambda }{1+\lambda }\right)}^{i+a-2}\frac{{\left(-1\right)}^{i+j}{\left(1-\delta \right)}^{k}e^{i(1+\lambda )}}{{\left(i\lambda \right)}^{i+a}}\\ & & \times \{\Gamma (i+a,i(1+\lambda ))-\Gamma (i+a,i(1+\lambda +\lambda t))\}, \end{array}$$ (18)

For a continuous distribution with pdf *g*(*t*) and CDF *G*(*t*), the strong mean inactivity time (SMIT) function is defined as

$$\begin{array}{c} m^{*}(t)=\frac{1}{G(t)}\int_{0}^{t}2xG\left(x\right)dx.\;t>0. \end{array}$$ (19)

For the EGLD, the strong mean inactivity time function m* is

$$\begin{array}{ccc} m^{*}(t) & = & \frac{2\kappa^{\gamma }}{1-\left(1-\delta \right)\left(1-\kappa^{\gamma }\right)}(D(t;\Lambda ,2)-D(t;\Lambda ,1)). \end{array}$$ (20)

**Fig 3:**
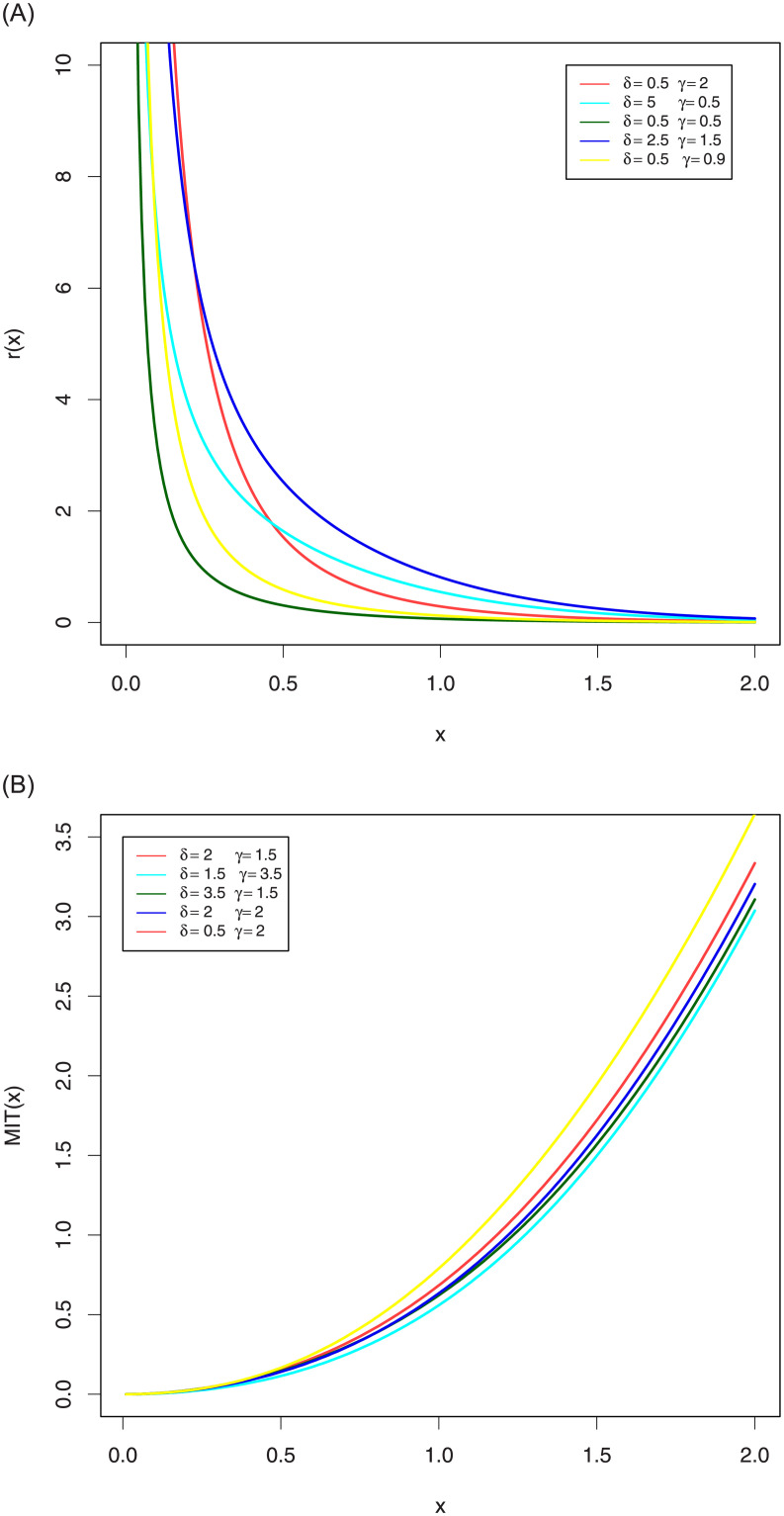
Plots RHR and MIT of the EGLD with λ = 2.9. Figure (3) shows the different shapes of its RHR and MIT for some selected parameters values with scale parameter one. This Figure indicates that the EGLD RHR can be monotonically decreasing and MIT can be monotonically increasing.

Fig (3) shows the different shapes of its RHR and MIT for some selected parameters values with scale parameter one. This Figure indicates that the EGLD RHR can be monotonically decreasing and MIT can be monotonically increasing.

### 3.3 Renyi entropy

Entropy has been used in areas like physics (sparse kernel density estimation), medicine (molecular imaging of tumors) and engineering (measure the randomness of systems). The entropy is a measure of variation of the uncertainty of a random variable *X* with density function *f*(*x*). The Rényi entropy (RE) [16] of order *b* is defined as

$$\begin{array}{c} H_{b}=\frac{1}{1-b}\log(\int_{-\infty }^{\infty }g{\left(x\right)}^{b}dx),\;b>0,b\neq 1. \end{array}$$ (21)

For the EGLD(*x*;Λ) in (6) can be obtained as

$$\begin{array}{ccc} H_{b} & = & \frac{b}{1-b}\log(\frac{\lambda^{2}\gamma }{\delta (\lambda +1)})+\frac{1}{1-b}\log\{\sum_{j=0}^{\infty }{\left(\frac{\delta -1}{\delta }\right)}^{j}\frac{1+\lambda }{\lambda }C_{0,b,b}\left(\lambda ,\gamma \right)\}. \end{array}$$

Table 1 present the critical points of the reliability functions of the EGLD. These values can be determined numerically using R and Maple^14^.

**Table 1:** Critical points of reliability functions for selected values of λ = 2.90 and *γ* = 3.00 at *t* = 0.5.

*δ*	030	0.50	0.65	0.80	0.95	1.00
HR	2.93562	2.31981	2.00445	1.76457	1.57597	1.52176
MRL	0.354484	0.398896	0.428236	0.454937	0.479475	0.487237
RHR	1.94233	2.55815	2.8735	3.11338	3.30198	3.3562
MIT	0.2035	0.18559	0.178116	0.173002	0.169276	0.168248
SMIT	0.149169	0.138466	0.133869	0.130681	0.128335	0.127684
Renyi entropy	0.625869	0.739825	0.794179	0.835126	0.867617	0.877068

From Table 1 we have the following observations:
For fixed λ and *γ*, the HR, MIT and SMIT of the different parameters decrease as *δ* increases. For fixed λ and *γ*, the MRL and RHR of the different parameters increases as *δ* increases.

### 3.4 Quantiles and moments

**Lemma 3.3**. *Let X ∼ EGLD* (*x*;Λ). *Then, the qth quantile function, denoted bt* (*x*_*q*_)_*EGL*_, *is given by*

$$\begin{array}{c} {\left(x_{q}\right)}_{EGL}=\ln{\left[\frac{1+\frac{\lambda }{1+\lambda }{\left(x_{q}\right)}_{EGL}}{1-{\left(\frac{\delta q}{1-q(1-\delta )}\right)}^{\frac{1}{\gamma }}}\right]}^{\frac{1}{\lambda }}. \end{array}$$ (22)

**Remark 3.4**

- *The median of the EGLD* (*x*;Λ) *as*
  $$\begin{array}{c} Med_{GLE}\left(X\right)=\ln{\left[\frac{1+\frac{\lambda }{1+\lambda }Med_{GLE}\left(X\right)}{1-{\left(\frac{\delta }{1+\delta }\right)}^{\frac{1}{\gamma }}}\right]}^{\frac{1}{\lambda }}. \end{array}$$ (23)
- *The qth quantiles of the GL* (*x*;λ, *γ*) *model as*
  $$\begin{array}{c} Med_{GL}\left(X\right)=\ln{\left[\frac{1+\frac{\lambda }{1+\lambda }Med_{GL}\left(X\right)}{1-{\left(\frac{1}{2}\right)}^{\frac{1}{\gamma }}}\right]}^{\frac{1}{\lambda }}. \end{array}$$ (24)
- *The qth quantiles of the L*(*x*;λ) *model as*
  $$\begin{array}{c} Med_{L}\left(X\right)=\ln{\left[2(1+\frac{\lambda }{1+\lambda }Med_{L}\left(X\right))\right]}^{\frac{1}{\lambda }}. \end{array}$$ (25)

The next lemma are need in the noncentral moment of the EGLD.

**Lemma 3.5**. *For* λ > 0 *and γ* > 0. *Let*

$$\begin{array}{ccc} C_{r,u,p}\left(\lambda ,\gamma \right) & = & \frac{\lambda }{1+\lambda }\int_{0}^{\infty }x^{r}{\left(1+x\right)}^{p}e^{-u\lambda x}{\left[1-\frac{1+\lambda +\lambda x}{1+\lambda }e^{-\lambda x}\right]}^{\gamma (k+u)-u}dx, \end{array}$$

*we have*

$$\begin{array}{ccc} C_{r,u,p}\left(\lambda ,\gamma \right) & = & \sum_{j=0}^{\infty }\sum_{i=j}^{\infty }\sum_{l=0}^{p}{\left(-1\right)}^{i}(\begin{array}{c} \gamma (k+u)-u\\ i \end{array})(\begin{array}{c} i\\ j \end{array})(\begin{array}{c} p\\ l \end{array}){\left(\frac{\lambda }{1+\lambda }\right)}^{j+1}\\ & & \times \frac{\Gamma (r+j+l+1)}{{\left(u\lambda (j+1)\right)}^{r+j+l+1}}. \end{array}$$

**Proposition 3.6**. *Let X*∼ *EGLD* (*x*;Λ). *Then, the noncentral moment of X has the following form*:

$$\begin{array}{ccc} \mu_{r}^{{}^{\prime }} & = & \frac{\lambda \gamma }{\delta }\sum_{k=0}^{\infty }{\left(\frac{\delta -1}{\delta }\right)}^{k}\left(k+1\right)C_{r,1,1}\left(\lambda ,\gamma \right) \end{array}$$ (26)

*assuming that* Λ > 0.

Based on proposition (3.6), the following measures hold for every Λ > 0 of the EGLD (*x*;Λ).

$$\begin{array}{c} \mu_{2}\left(x;\Lambda \right)=\frac{\lambda \gamma }{\delta }\sum_{k=0}^{\infty }{\left(\frac{\delta -1}{\delta }\right)}^{k}\left(k+1\right)C_{2,1,1}\left(\lambda ,\gamma \right), \end{array}$$ (27)

and the variance of the EGLD (*x*;Λ) as

$$\begin{array}{c} \sigma^{2}=\frac{\lambda \gamma }{\delta }\sum_{k=0}^{\infty }{\left(\frac{\delta -1}{\delta }\right)}^{k}\left(k+1\right)\{C_{2,1,1}\left(\lambda ,\gamma \right)-C_{1,1,1}^{2}\left(\lambda ,\gamma \right)\}. \end{array}$$ (28)

The measures of skewness and kurtosis are computed using the following expressions:

$$\begin{array}{ccc} \text{Skewness} & = & \frac{\mu_{3}^{{}^{\prime }}-3\mu_{2}^{{}^{\prime }}\mu +2\mu^{3}}{{\left(\mu_{2}^{{}^{\prime }}-\mu^{2}\right)}^{\frac{3}{2}}},\\ \text{Kurtosis} & = & \frac{\mu_{4}^{{}^{\prime }}-4\mu_{3}^{{}^{\prime }}\mu +6\mu_{2}^{{}^{\prime }}\mu^{2}-3\mu^{4}}{{\left(\mu_{2}^{{}^{\prime }}-\mu^{2}\right)}^{2}}. \end{array}$$

Table 2 lists the first six moments, variance, skewness and kurtosis for the EGLD (*x*;Λ) for some selected values for *δ* by choosing the scale and shape parameters to be one in all cases.

**Table 2:** Moments of the EGLD for some parameter values, λ = 2.90 and *γ* = 3.00.

$\mu_{r}^{{}^{\prime }}$	*δ* = 0.30	*δ* = 0.65	*δ* = 0.80	*δ* = 1.00
$\mu_{1}^{{}^{\prime }}$	0.518682	0.679073	0.727784	0.782748
$\mu_{2}^{{}^{\prime }}$	0.40514	0.65132	0.735537	0.835731
$\mu_{3}^{{}^{\prime }}$	0.450433	0.828127	0.968904	1.1432
$\mu_{4}^{{}^{\prime }}$	0.669297	1.33013	1.59089	1.92291
$\mu_{5}^{{}^{\prime }}$	1.2536	2.59797	3.1476	3.86051
$\mu_{6}^{{}^{\prime }}$	2.82781	5.98913	7.30897	9.04088
Variance	0.13611	0.190179	0.205868	0.223037
Skewness	1.97349	1.53782	1.4339	1.32789
Kurtosis	9.26325	6.76956	6.28049	5.82284

From Table 2 we have the following observations:
For fixed λ and *γ*, the Skewness, and Kurtosis of the different parameters decrease as *δ* increases. For fixed λ and *γ*, the moments and Variance of the different parameters increases as *δ* increases.

### 3.5 Order statistics

Order statistics have various applications in many different areas of statistical theories and applications such as quality control testing and reliability. Let *X*_1_, …, *X*_*n*_ be a random sample of size *n* from the EGLD (*x*;Λ). The PDF of the *i*^*th*^ order statistic, *X*_*i*: *n*_, is defined by

$$\begin{array}{c} f_{i:n}\left(x\right)=\frac{\lambda^{2}\gamma \delta^{n-i+1}}{\left(1+\lambda )B(i,n-i+1\right)}\frac{\kappa^{i-\frac{1}{\gamma }}{\left(1-\kappa \right)}^{n-i}}{{\left(\delta +\bar{\delta }\kappa \right)}^{n+1}} \end{array}$$ (29)

where *B*(.,.) is the beta function. Also, the joint pdf of the (*i*, *j*)^*th*^ order statistic, *X*_*i*: *n*_, *X*_*j*: *n*_ and 1 ≤ *i* ≤ *j* ≤ *n*, is defined by

$$\begin{array}{ccc} f_{i:j:n}\left(x_{i},x_{j}\right) & = & \varepsilon {\left[F(x_{i})\right]}^{i-1}{\left[F\left(x_{j}\right)-F\left(x_{i}\right)\right]}^{j-i-1}{\left[1-F(x_{j})\right]}^{n-j}f\left(x_{i}\right)f\left(x_{j}\right)\\ & = & \varepsilon {\left(\frac{\lambda^{2}\gamma }{1+\lambda }\right)}^{2}\frac{\delta^{n-i+1}{\left(\kappa_{i}\kappa_{j}\right)}^{1-\frac{1}{\gamma }}{\left(1-\kappa_{j}\right)}^{n-j}{\left(\kappa_{j}-\kappa_{i}\right)}^{j-i-1}}{{\left(\delta +\bar{\delta }\kappa_{i}\right)}^{j}{\left(\delta +\bar{\delta }\kappa_{j}\right)}^{n-i+1}}\\ & & \times \left(1+x_{i}\right)\left(1+x_{j}\right)e^{-(x_{i}+x_{j})\lambda }, \end{array}$$ (30)

where $\kappa_{s}={\left(1-\frac{1+\lambda +\lambda x_{s}}{\lambda +1}e^{-\lambda x_{s}}\right)}^{\gamma }$ and $\varepsilon =\frac{n!}{(i-1)!(j-i-1)!(n-j)!}.$

### 3.6 Stochastic orderings

Stochastic orders has many applications in different fields such as income, actuarial science, wealth inequality, engineering, medical and biological sciences, lifetime, queuing theory and reliability analysis (Shaked and Shanthikumar [17]). Let *X*_1_ and *X*_2_ be univariate random variables with distribution functions *G*_1_(*x*) and *G*_2_(*x*) and reliability functions ${\bar{G}}_{1}\left(x\right)$ and ${\bar{G}}_{2}\left(x\right)$, respectively, with corresponding probability densities *g*_1_(*x*), *g*_2_(*x*).

- If *G*_1_(*x*)≥*G*_2_(*x*), ∀*x*, then *X*_1_≤_*st*_ *X*_2_ (stochastically ordering).
- If *g*_1_(*x*)≥*g*_2_(*x*), ∀*x*, then *X*_1_≤_*lr*_ *X*_2_ (likelihood ratio ordering).
- If *h*_1_(*x*)≥*h*_2_(*x*), ∀*x*, then *X*_1_≤_*hr*_ *X*_2_ (hazard rate ordering).
- If *m*_1_(*x*)≥*m*_2_(*x*), ∀*x*, then *X*_1_≤_*mrl*_ *X*_2_ (mean residual life ordering).
- If *G*_1_(*x*)/*G*_2_(*x*) is decreasing, ∀*x*, then *X*_1_≤_*rhr*_ *X*_2_ (reversed hazard rate ordering).

From the last stochastic orders, the following implications are satisfied (Shaked and Shanthikumar [17]):

$$\begin{array}{c} X_{1}\leq_{rhr}X_{2}⇐X_{1}\leq_{lr}X_{2}\Rightarrow X_{1}\leq_{hr}X_{2}\Rightarrow X_{1}\leq_{st}X_{2}\Rightarrow X_{1}\leq_{mrl}X_{2}. \end{array}$$ (31)

The next theorem propose the EGLD are ordered with respect to the strongest likelihood ratio ordering when suitable assumptions are satisfied.

**Theorem 3.7**. *Let X and Y be univariate random variables such that X* ∼ *EGLD*(λ, *γ*, *δ*_1_) *and Y* ∼ *EGLD*(λ, *γ*, *δ*_2_) *If δ*_1_ < *δ*_2_, *then*

$$\begin{array}{c} X\leq_{lr}Y\left(X\leq_{hr}Y,X\leq_{rhr}Y,X\leq_{st}Y\right). \end{array}$$ (32)

## 4 Maximum likelihood estimates

Let *x* = (*x*_1_, *x*_2_, …, *x*_*n*_) of a random sample of size *n* from EGLD with three parameters (Λ = (λ, *γ*, *δ*)). The log-likelihood function (LLF) takes the form

$$\begin{array}{ccc} \psi (X_{1},...,X_{n}|\delta ) & = & \sum_{i=1}^{n}\log\left(x_{i}+1\right)-\sum_{i=1}^{n}\log(-\lambda +\lambda \left(-x_{i}\right)+\left(\lambda +1\right)e^{\lambda x_{i}}-1)\\ & & -2\sum_{i=1}^{n}\log(\delta +\left(1-\delta \right){\left(1-\frac{\left(\lambda +\lambda x_{i}+1\right)e^{-\lambda x_{i}}}{\lambda +1}\right)}^{\gamma })+n\log\left(\delta \right)\\ & & +n\log\left(\gamma \right)+2n\log\left(\lambda \right)+\gamma \sum_{i=1}^{n}\log(1-\frac{\left(\lambda +\lambda x_{i}+1\right)e^{-\lambda x_{i}}}{\lambda +1}). \end{array}$$ (33)

The MLEs of the unknown parameters λ, *γ* and *δ* can be obtained by solving the

$$\begin{array}{ccc} \Psi_{\lambda } & = & \frac{\partial \psi }{\partial \lambda }=\frac{2n}{\lambda }-\sum_{i=1}^{n}\frac{x_{i}(\left(\lambda +1\right)e^{\lambda x_{i}}-1)+e^{\lambda x_{i}}-1}{\left(\lambda +1\right)(e^{\lambda x_{i}}-1)-\lambda x_{i}}\\ & & +\sum_{i=1}^{n}\frac{{\left(\dot{v_{i}}\right)}_{\lambda }}{v_{i}}-2\sum_{i=1}^{n}\frac{\left(\delta -1\right){\left(\dot{v_{i}}\right)}_{\lambda }}{\left(\delta -1\right)v_{i}-\delta }, \end{array}$$ (34)

$$\Psi_{\gamma }=\frac{\partial \psi }{\partial \gamma }=\frac{n}{\gamma }+\sum_{i=1}^{n}\frac{{\left(\dot{v_{i}}\right)}_{\gamma }}{v_{i}}-2\sum_{i=1}^{n}\frac{\left(1-\delta \right){\left(\dot{v_{i}}\right)}_{\gamma }}{\left(1-\delta \right)v_{i}+\delta },$$ (35)

$$\Psi_{\delta }=\frac{\partial \psi }{\partial \delta }=\frac{n}{\delta }-2\sum_{i=1}^{n}\frac{1-v_{i}}{\delta +\left(1-\delta \right)v_{i}}.$$ (36)

These equations can be solved numerically by using statistical software. The asymptotic confidence intervals (CIs) for the parameters of EGLD(Λ) distribution are given according to the asymptotic distribution of the maximum likelihood estimates (MLEs) of the parameters. For more details about the maximum likelihood estimates see for example Dong et al. [18], Chen et al. [19] and Chen et al. [20]. The second derivatives of the LLF of EGLD with respect to Λ are given in the Appendix part [**B**], from Eqs (41)–(46). The estimators are approximately bi-variate normal with mean Λ and the observed information matrix is given by

$$\begin{array}{c} I_{n}\left(\Lambda \right)=-\frac{\partial^{2}\psi }{\partial \Lambda \partial \Lambda }=-{\left[\begin{array}{ccccc} \Psi_{\lambda \lambda } & & \Psi_{\lambda \gamma } & & \Psi_{\lambda \delta }\\ \Psi_{\gamma \lambda } & & \Psi_{\gamma \gamma } & & \Psi_{\gamma \delta }\\ \Psi_{\delta \delta } & & \Psi_{\delta \gamma } & & \Psi_{\delta \delta } \end{array}\right]}_{\lambda =\hat{\lambda },\gamma =\hat{\gamma },\delta =\hat{\delta }} \end{array}$$ (37)

The 100(1−*ϑ*)% approximate two-sided confidence intervals (CIs) for the parameters λ, *γ* and *δ* are

$$\hat{\lambda }\pm Z_{\frac{\vartheta }{2}}\sqrt{Var(\hat{\lambda })},\;\hat{\gamma }\pm Z_{\frac{\vartheta }{2}}\sqrt{Var(\hat{\gamma })}\;\;\text{and}\;\;\hat{\delta }\pm Z_{\frac{\vartheta }{2}}\sqrt{Var(\hat{\delta })}$$

respectively, where $Z_{\frac{\vartheta }{2}}$ is the upper $(\frac{\vartheta }{2})th$ percentile of the standard normal distribution and $Var(\hat{\lambda })$, $Var(\hat{\gamma })$ and $Var(\hat{\delta })$ are given by the diagonal elements of *I*^−1^(Λ) and it’s called the variance of $\hat{\lambda }$, $\hat{\gamma }$ and $\hat{\delta }$.

## 5 Simulation study

In this section, a simulation study by considering different parameters values and different samples sizes is conducted to decide which estimation method provides the best estimates in terms of minimum mean square error (MSE). The samples sizes are selected to be 30, 50, 70, 100, 150 and the parameters values are selected to be (λ, *γ*, *δ*) = (2.90, 3.00, 0.70)*and*(0.20, 2.70, 0.90). The process is replicated 1000 times for each setting and the average estimates,the average Bias and the average MSEs are computed. For more details about the MSEs see for example Zeng et al. [21], Zeng et al. [22], Zeng et al. [23] and Zeng et al. [24]. These values are tabulated in Table 3. The results in these tables show that the four estimation methods provide an asymptotically unbiased estimates where the estimates tend to the true parameters values as the sample size increases. Also it is noted that the MSEs decreases in all the cases for the different estimates as the sample size increases. In addition, the simulation results shows that the LSEs have the smallest MSEs in most of the cases.

**Table 3:** Simulated average estimates (AEs) (first row), mean squared errors (MSEs) (second row) and bias estimates (third row) under different settings parameters.

EGLD(λ, *γ*, *δ*)	*n*	Estimates	λ	*γ*	*δ*
EGLD(2.90,3.00,0.70)	30	Est.	3.32791	2.96355	1.21411
		MSE	2.74349	1.89877	2.61318
		Bais	0.42791	-0.03645	0.51411
	50	Est.	3.19317	2.82378	1.13280
		MSE	0.92870	1.12008	1.93396
		Bais	0.29317	-0.17622	0.43280
	70	Est.	3.06135	2.92424	0.96370
		MSE	0.65713	0.84221	1.01533
		Bais	0.16135	-0.07576	0.26370
	100	Est.	3.04659	2.86435	0.93394
		MSE	0.48586	0.58779	0.73386
		Bais	0.14660	0.13565	0.23394
	150	Est.	2.99243	2.95314	0.84701
		MSE	0.30126	0.36852	0.32648
		Bais	0.09243	0.04687	0.14709
EGLD (0.20,2.70,0.90)	50	Est.	0.21920	2.63306	1.71376
		MSE	0.00315	0.75398	3.20126
		Bais	0.01920	-0.06694	0.81376
	100	Est.	0.21147	2.61271	1.40693
		MSE	0.00191	0.37813	1.45407
		Bais	0.01147	-0.08729	0.50693
	150	Est.	0.20638	2.68321	1.21072
		MSE	0.00101	0.24497	0.79587
		Bais	0.0064	-0.01679	0.31072

## 6 Application: Waiting time

The next data set studied the service of 100 bank customers and waiting times (in minutes). These data were considered by Ghitany et al. [25] and given by Table 4.

**Table 4:** The data set of waiting time presented.

0.8	0.8	1.3	1.5	1.8	1.9	1.9	2.1	2.6	2.7	2.9	3.1	3.2	3.3	3.5
3.6	4.0	4.1	4.2	4.2	4.3	4.3	4.4	4.4	4.6	4.7	4.7	4.8	4.9	4.9
5.0	5.3	5.5	5.7	5.7	6.1	6.2	6.2	6.2	6.3	6.7	6.9	7.1	7.1	7.1
7.1	7.4	7.6	7.7	8.0	8.2	8.6	8.6	8.6	8.8	8.8	8.9	8.9	9.5	9.6
9.7	9.8	10.7	10.9	11.0	11.0	11.1	11.2	11.2	11.5	11.9	12.4	12.5	12.9	13.0
13.1	13.3	13.6	13.7	13.9	14.1	15.4	15.4	17.3	17.3	18.1	18.2	18.4	18.9	19.0
19.9	20.6	21.3	21.4	21.9	23.0	27.0	31.6	33.1	38.5.					

To show the applicability of the proposed distribution and the different estimators presented in the previous sections one real data set is analyzed and shows the significance of our new distribution. We compare the results of the EGLD with GL and L distributions. We first use the maximum likelihood method to estimate the unknown parameters of the competitive distributions. These estimates are displayed in Table 5.

**Table 5:** The estimates of parameters corresponds to data set.

Distribution	parameter MLEs		
	$\hat{\lambda }$	$\hat{\gamma }$	$\hat{\delta }$
EGLD	0.172494	1.46019	0.461444
GLD	0.210779	1.27729	-
LD	0.186571	-	-

The observed information of the data and the asymptotic covariance matrix of MLEs, respectively, are

$$\begin{array}{c} I_{0}\left(\hat{\lambda },\hat{\gamma },\hat{\delta }\right)=\begin{array}{c} (\begin{array}{ccc} 7510.98 & -578.926 & -1036.28\\ -578.926 & 70.0194 & 89.0158\\ -1036.28 & 89.0158 & 155.088 \end{array}), \end{array} \end{array}$$ (38)

and

$$\begin{array}{c} I_{0}^{-1}\left(\hat{\lambda },\hat{\gamma },\hat{\delta }\right)=(\begin{array}{ccc} 0.00174401 & -0.00146198 & 0.0124924\\ -0.00146198 & 0.0540601 & -0.0407976\\ 0.0124924 & -0.0407976 & 0.113337 \end{array}). \end{array}$$ (39)

Therefor, 95% two-sided asymptotic confidence intervals for the parameters λ, *γ*, and *δ* respectively, are [0.161736,0.183251], [1.40029, 1.52008] and [0.374721, 0.548166]. Some goodness of fit measures are displayed in Table 6. From this table we can note the following:

- According to maximum log-likelihood criterion for goodness of fit and −*logL*, the order of best fit for the above models is: **Best** EGLD ⇒ GLD ⇒ LD **Worst**.
- To compare the different models with the EGLD we obtain the Kolmogorov-Smirnov (K-S) statistic as well as its p-value. These statistics are displayed also in Table 7 for the data set. From these results, we can conclude that the EGLD has the K-S value 0.040985 and the highest p-value 0.956357 among all other competitive models, therefore it can be selected as the best model.
- According to A and W, the order of best fit for the above models is: **Best** GLD ⇒ LD ⇒ EGLD **Worst**.
- According to these statistics, the EGLD model fits the current data set better than the other models.

**Table 6:** Goodness-of-fit statistics corresponds to data set.

Distribution	−*logL*	*K*−*S*	*A*	*W*
		(*p*-value)	(*p*-value)	(*p*-value)
EGLD	317.155	0.040985	0.129516	0.0185372
		(0.956357)	(0.991705)	(0.982528)
GLD	317.803	0.0471189	0.240452	0.037585
		(0.855563)	(0.784184)	(0.721155)
LD	319.037	0.0495455	0.267223	0.0419412
		(0.799012)	(0.706235)	(0.645525)

**Table 7:** Likelihood ratio test and its p-value corresponds to data set.

Distribution	Null hypothesis (*H*_0_)	Alternative hypothesis (*H*_1_)	LRT	(*p*-value)
GL	*H*_0_: *δ* = 1(*GL*)	*H*_1_: *δ* ≠ 1(EGLD)	19.5512	9.79415×10^−6^ < 0.05)
L	*H*_0_: *δ* = 1, *γ* = 1(*L*)	*H*_1_: *δ* ≠ 1, *γ* ≠ 1(EGLD)	4.9492	0.0261028 < 0.05)

In order to see how well the EGLD fits this data, we introduce the hypotheses test statistic as well as its p-value. The hypotheses are as follows:

$$H_{0}:F=F_{\text{EGLD}}\;\text{versus}\;H_{1}:F\neq F_{\text{EGLD}}.$$

**Fig 4:**
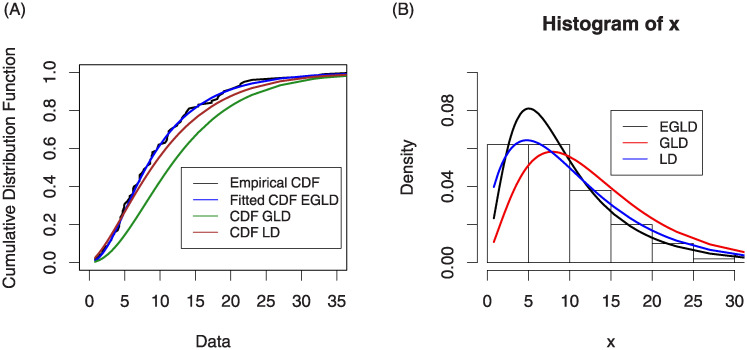
Plots estimated CDF and PDF of models for the data set. Figure (4) shows plots of the estimated cumulative and estimated densities of the fitted models for the data data described below.

Furthermore, likelihood ratio test (LRT) has been used to determine the appropriateness of the model. The hypotheses are as follows:

According to these statistics, the calculated LRT statistic is greater than the critical point for this test, which is 9.210; also, the *p*-value is small. furthermore, we conclude that this data fits the EGLD much better the GL and L distributions. Fig (4) shows plots of the estimated cumulative and estimated densities of the fitted models for the data data described below.

## Concluding remarks

Introducing a new model of the EGLD is the main goal of this article. This model has the characteristic of being capable of failure criteria and modeling various shapes of aging. The proposed distribution contains one scale and two shape parameters. The distributions GL, L and among others are sub-models of the EGLD and studied in this article. Some statistical properties of the new distribution are discussed. estimation methods are used to estimate the unknown parameters of the proposed distribution. The efficiency of the different estimators are compared via simulation study in terms of minimum mean square errors. The simulation study shows that the least square estimates perform better than other proposed methods. Finally, two real data sets are analyzed showing that the new distribution is very competitive as compared to some well known distribution with three or more than three parameters. A future work is to estimate procedures of stress-strength reliability for Generalized Lindley Distribution. Another future work is to study and compare the Bayesian estimation based on maximum likelihood and based on maximum product of spacing to estimate the stress-strength reliability of Generalized Lindley Distribution.

## Appendix

**[A] Proofs of lemma and theorem**.

**Proof of Lemma 3**.**5**

$$\begin{array}{ccc} C_{r,u,p}\left(\lambda ,\gamma \right) & = & \frac{\lambda }{1+\lambda }\int_{0}^{\infty }x^{r}{\left(1+x\right)}^{p}e^{-u\lambda x}{\left[1-\frac{1+\lambda +\lambda x}{1+\lambda }e^{-\lambda x}\right]}^{\gamma (k+u)-u}dx\\ & = & \frac{\lambda }{1+\lambda }\sum_{i=0}^{\infty }(\begin{array}{c} \gamma (k+u)-u\\ i \end{array}){\left(-1\right)}^{i}\int_{0}^{\infty }x^{r}{\left(1+x\right)}^{p}e^{-(u+i)\lambda x}\\ & & \times {\left[1+\frac{\lambda }{1+\lambda }x\right]}^{i}dx\\ & = & \sum_{i=0}^{\infty }\sum_{j=0}^{i}\sum_{l=0}^{p}{\left(-1\right)}^{i}(\begin{array}{c} \gamma (k+u)-u\\ i \end{array})(\begin{array}{c} i\\ j \end{array})(\begin{array}{c} p\\ l \end{array}){\left(\frac{\lambda }{1+\lambda }\right)}^{j+1}\\ & & \times \int_{0}^{\infty }x^{r+j+l}e^{-u\lambda (j+1)x}dx.\\ & = & \sum_{i=0}^{\infty }\sum_{j=0}^{i}\sum_{l=0}^{p}{\left(-1\right)}^{i}(\begin{array}{c} \gamma (k+u)-u\\ i \end{array})(\begin{array}{c} i\\ j \end{array})(\begin{array}{c} p\\ l \end{array}){\left(\frac{\lambda }{1+\lambda }\right)}^{j+1}\\ & & \times \frac{\Gamma (r+j+l+1)}{{\left(u\lambda (j+1)\right)}^{r+j+l+1}}. \end{array}$$

By changing $\sum_{i=0}^{\infty }\sum_{j=0}^{i}$ to $\sum_{j=0}^{\infty }\sum_{i=j}^{\infty }$, in the last equation and hence, the proof is completed.

**Proof of Theorem (3.7)**

First note that

$$\begin{array}{c} \frac{f(x)}{g(x)}=\frac{\delta_{1}}{\delta_{2}}{\left[\frac{\delta_{2}-(\delta_{2}-1)v\left(x\right)}{\delta_{1}-(\delta_{1}-1)v\left(x\right)}\right]}^{2}. \end{array}$$ (40)

Since, *δ*_1_ < *δ*_2_,

$$\begin{array}{ccc} \frac{d}{dx}[\frac{f(x)}{g(x)}] & = & \frac{2\lambda^{2}\gamma \delta_{1}(\delta_{2}-\delta_{1})\left(x+1\right)v\left(x\right)((\delta_{2}-1)v\left(x\right)-\delta_{2})}{\delta_{2}(\lambda +\lambda x-\left(\lambda +1\right)e^{\lambda x}+1){\left((\delta_{1}-1)v\left(x\right)-\delta_{1}\right)}^{3}}\\ & < & 0. \end{array}$$

where

$$\begin{array}{ccc} v(x) & = & {\left(1-\frac{\left(\lambda +\lambda x+1\right)e^{-\lambda x}}{\lambda +1}\right)}^{\gamma }. \end{array}$$

Hence, *f*(*x*)/*g*(*x*) is decreasing in *x*. That is *X*≤_*lr*_ *Y*. The remaining statements follows from the implications Eq (31).

**[B]** The entries of the FIM for EGL distribution with respect to λ, *γ* and *δ* are given by the following equations:

$$\begin{array}{ccc} & & \Psi_{\lambda \lambda }=\frac{\partial^{2}\psi }{\partial \lambda^{2}}=-\gamma \sum_{i=1}^{n}\frac{x_{i}^{2}(2\lambda +(\lambda^{2}+2\lambda -1){\left(\lambda +1\right)}^{2}e^{\lambda x_{i}}+1)+\lambda {\left(\lambda +1\right)}^{3}x_{i}^{2}e^{\lambda x_{i}}}{{\left(\lambda +1\right)}^{4}e^{2\lambda x_{i}}v_{i}^{\frac{2}{\gamma }}}\\ & & +\sum_{i=1}^{n}\frac{{\left(e^{\lambda x_{i}}-1\right)}^{2}+x_{i}(e^{\lambda x_{i}}\left(x_{i}\left(\lambda \left(\lambda +2\right)+\lambda \left(\lambda +1\right)x_{i}-1\right)-2\right)+x_{i}+2)}{{\left(\lambda +1\right)}^{2}e^{2\lambda x_{i}}v_{i}^{\frac{2}{\gamma }}}\;\\ & & -2\sum_{i=1}^{n}\frac{\gamma \left(1-\delta \right)x_{i}v_{i}^{\frac{\gamma -2}{\gamma }}e^{-2\lambda x_{i}}}{{\left(\lambda +1\right)}^{4}{\left(\delta +\delta \left(-v_{i}\right)+v_{i}\right)}^{2}}\{\lambda^{2}\gamma \left(\delta -1\right)v_{i}x_{i}{\left(\lambda +\left(\lambda +1\right)x_{i}+2\right)}^{2}\\ & & -\lambda^{2}\left(\gamma -1\right)x_{i}\left(\left(\delta -1\right)v_{i}-\delta \right){\left(\lambda +\left(\lambda +1\right)x_{i}+2\right)}^{2}+\left(\left(\delta -1\right)v_{i}-\delta \right)\\ & & \times (\left(\lambda +1\right)(e^{\lambda x_{i}}-1)-\lambda x_{i})x_{i}(\lambda^{3}+3\lambda^{2}+\lambda +{\left(\lambda +1\right)}^{2}\lambda x_{i}-1)-2\}\\ & & -\gamma \sum_{i=1}^{n}\frac{2\left(\lambda +1\right)(e^{\lambda x_{i}}-1)}{{\left(\lambda +1\right)}^{4}e^{2\lambda x_{i}}v_{i}^{\frac{2}{\gamma }}}-\frac{2n}{\lambda^{2}}, \end{array}$$ (41)

$$\Psi_{\gamma \gamma }=\frac{\partial^{2}\psi }{\partial \gamma^{2}}=2\sum_{i=1}^{n}\frac{\left(\delta -1\right)\delta {\left[{\left(\dot{v_{i}}\right)}_{\gamma }\right]}^{2}}{v_{i}{\left(\delta -\left(\delta -1\right)v_{i}\right)}^{2}}+\frac{n}{\gamma^{2}},$$ (42)

$$\Psi_{\delta \delta }=\frac{\partial^{2}\psi }{\partial \delta^{2}}=2\sum_{i=1}^{n}\frac{{\left(1-v_{i}\right)}^{2}}{{\left(\delta +\left(1-\delta \right)v_{i}\right)}^{2}}+\frac{n}{\delta^{2}},$$ (43)

$$\Psi_{\lambda \gamma }=\frac{\partial^{2}\psi }{\partial \lambda \partial \gamma }=\frac{1}{\gamma }\sum_{i=1}^{n}\frac{{\left(\dot{v_{i}}\right)}_{\lambda }}{v_{i}}+\frac{2}{\gamma }\sum_{i=1}^{n}\frac{{\left(\dot{v_{i}}\right)}_{\lambda }(\delta -\left(\delta -1\right)v_{i}+\gamma \delta \frac{{\left(\dot{v_{i}}\right)}_{\gamma }}{v_{i}})}{{\left(\delta -\left(\delta -1\right)v_{i}\right)}^{2}},$$ (44)

$$\Psi_{\lambda \delta }=\frac{\partial^{2}\psi }{\partial \lambda \partial \delta }=2\sum_{i=1}^{n}\frac{{\left(\dot{v_{i}}\right)}_{\lambda }}{{\left(\delta -\left(\delta -1\right)v_{i}\right)}^{2}},$$ (45)

$$\Psi_{\gamma \delta }=\frac{\partial^{2}\psi }{\partial \gamma \partial \delta }=2\sum_{i=1}^{n}\frac{{\left(\dot{v_{i}}\right)}_{\gamma }}{{\left(\delta -\left(\delta -1\right)v_{i}\right)}^{2}},$$ (46)

where

$$\begin{array}{ccc} v_{i} & = & v_{i}\left(\lambda ,\gamma ,\delta \right)={\left(1-\frac{\left(\lambda +\lambda x_{i}+1\right)e^{-\lambda x_{i}}}{\lambda +1}\right)}^{\gamma },\\ {\left(\dot{v_{i}}\right)}_{\lambda } & = & -\frac{\lambda \gamma x_{i}\left(\lambda +\lambda x_{i}+x_{i}+2\right){\left(1-\frac{e^{\lambda (-x_{i})}\left(\lambda +\lambda x_{i}+1\right)}{\lambda +1}\right)}^{\gamma }}{\left(\lambda +1\right)(\lambda +\lambda x_{i}-\left(\lambda +1\right)e^{\lambda x_{i}}+1)}\\ {\left(\dot{v_{i}}\right)}_{\gamma } & = & {\left(1-\frac{e^{\lambda (-x_{i})}\left(\lambda +\lambda x_{i}+1\right)}{\lambda +1}\right)}^{\gamma }\log(1-\frac{e^{\lambda (-x_{i})}\left(\lambda +\lambda x_{i}+1\right)}{\lambda +1}) \end{array}$$

## References

[1] Lindley D.V. Fiducial distributions and Bayes theorem. J R Stat Soc. 1958, 20(1), 102–107.

[2] Zakerzadeh H.; Dolati A. Generalized Lindley distribution. J Math Extension. 2009, 3, 13–25.

[3] Oluyede B.; Yang T. A new class of generalized Lindley distributions with applications. J. Stat. Comput. Simul. 2015, 85, 2072–2100. 10.1080/00949655.2014.917308

[4] Nadarajah S.; Bakouch H.S.; Tahmasbi R. A generalized Lindley distribution. Sankhya B. 2011, 73, 331–359. 10.1007/s13571-011-0025-9

[5] Marshall A.W.; Olkin I. A new method for adding a parameter to a family of distributions with application to the exponential and Weibull families. Biometrika. 1997, 84, 641–652. 10.1093/biomet/84.3.641

[6] Ghitany M.E.; AL-Hussaini E.K.; AL Jarallah R.A. Marshall-Olkin extended Weibull distribution and its application to censored data. J Appl Stat. 2005, 32, 1025–1034. 10.1080/02664760500165008

[7] Lemonte A.J. A new extension of the Birnbaum–Saunders distribution. Braz J Probab Stat. 2013, 27, 133–149. 10.1214/11-BJPS160

[8] Cordeiro G.M.; Lemonte A.J. On the Marshall-Olkin extended Weibull distribution. Stat Papers. 2013, 54, 333–353. 10.1007/s00362-012-0431-8

[9] Okasha H.M.; Kayid M. A new family of Marshall–Olkin extended generalized linear exponential distribution. J COMPUT APPL MATH. 2016, 296, 576–592. 10.1016/j.cam.2015.10.017

[10] Okasha H.M.; Ali A.A. Generalized Linear Exponential Geometric Distributions and its Applications. J COMPUT APPL MATH. 2019, 351, 198–211. 10.1016/j.cam.2018.10.041

[11] Okasha H.M.; El-Baz A.H.; Tarabia A.M.K.; Abdulkareem M.B. Extended inverse Weibull distribution with reliability application. J. Egyptian Math. Soc. 2017, 25, 343–349. 10.1016/j.joems.2017.02.006

[12] Benkhelifa L. The Marshall–Olkin extended generalized Lindley distribution: Properties and applications. COMMUN STAT-SIMUL C. 2017, 46(10), 8306–8330. 10.1080/03610918.2016.1277747

[13] Okasha HM, Shrahili M. A New Family of Marshall-Olkin Burr type XII Distribution. J COMPUT THEOR NANOS. 2017, 14, 5261–5269.

[14] Navarro J.; Franco M.; Ruiz J.M. Characterization through moments of the residual life and conditional spacing. The Indian Journal of Statistics, Series A. 1998, 60, 36–48.

[15] Wall H. Analytic Theory of continued fractions. New York: D. Van Nostrand, 1948.

[16] Rényi A. On measures of entropy and information. Proceedings of the 4th Berkeley Symposium on Mathematical Statistics and Probability, University of California Press, Berkeley. 1961, I, 547–561.

[17] Shaked M.; Shanthikumar J.G. Stochastic Orders. Springer: New York, 2007.

[18] Dong B.; Ma X.; Chen F.; Chen S. Investigating the differences of single-vehicle and multivehicle accident probability using mixed logit model. J ADV TRANSPORT. 2018 10.1007/s11116-016-9747-x.

[19] Chen F.; Chen S. Injury severities of truck drivers in single- and multi-vehicle accidents on rural highway. Accident Analysis and Prevention, 2011, 43(5), 1677–1688. 10.1016/j.aap.2011.03.026 21658494

[20] Chen F.; Chen S.; Ma X. Analysis of hourly crash likelihood using unbalanced panel data mixed logit model and real-time driving environmental big data. Journal of Safety Research. 2018; 65:153–159. 10.1016/j.jsr.2018.02.010 29776524

[21] Zeng Q.; Wen H.; Huang H.; Pei X.; Wong S.C. A multivariate random parameters Tobit model for analyzing highway crash rate by injury severity. Accid Anal Prev. 2017, 99, 184–191. 10.1016/j.aap.2016.11.018 27914307

[22] Zeng Q.; Guo Q.; Wong S.C.; Wen W. H.; Huang H.; Pei X. Jointly modeling area-level crash rates by severity: a Bayesian multivariate random-parameters spatio-temporal Tobit regression. TRANSPORTMETRICA A. 2019, 15(2), 1867–1884. 10.1080/23249935.2019.1652867

[23] Zeng Q.; Wen W. H.; Wong S.C.; Huang H.; Guo Q.; Pei X. Investigating the Impacts of Real-Time Weather Conditions on Freeway Crash Severity: A Bayesian Spatial Analysis. Int. J. Environ. Res. Public Health. 2020, 17(8), 27–68. 10.3390/ijerph17082768 32316427 PMC7215785

[24] Zeng Q.; Hao W.; Lee J.; Chen F. Spatial joint analysis for zonal daytime and nighttime crash frequencies using a Bayesian bivariate conditional autoregressive model. J. Transp. Saf. Secur. 2020, 12(4), 566–585. 10.1080/19439962.2018.1516259

[25] Ghitany M.E.; Atieh B.; Nadarajah S. Lindley distribution and its application. Math Comput Simul. 2008, 78(4), 493–506. 10.1016/j.matcom.2007.06.007

